# Species Distribution and Antifungal Susceptibility of Candida Isolates From Candidemia: Findings at a Tertiary Academic Medical Center From 2018 to 2025

**DOI:** 10.7759/cureus.115734

**Published:** 2026-09-03

**Authors:** Abdullah Kilic, Elizabeth L Palavecino

**Affiliations:** 1 Pathology, Wake Forest University, Winston-Salem, USA; 2 Pathology, Atrium Health Wake Forest Baptist, Winston-Salem, USA

**Keywords:** antifungal, candida, candidemia, species distribution, susceptibility

## Abstract

Background and objective

Candidemia remains a major cause of bloodstream infections, associated with substantial morbidity and mortality. Ongoing surveillance is needed because the distribution of *Candida* species and patterns of antifungal resistance continue to evolve. This study aimed to characterize the epidemiology and antifungal susceptibility profiles of *Candida* species isolated from blood cultures at a tertiary academic medical center from 2018 to 2025.

Methods

We conducted a retrospective laboratory-based analysis of non-duplicate *Candida* isolates recovered from positive blood cultures between January 2018 and December 2025. Species identification was performed using MALDI-TOF (matrix-assisted laser desorption/ionization time-of-flight) mass spectrometry. Antifungal susceptibility testing was conducted using Sensititre™ YeastOne™ (Thermo Fisher Scientific, Cleveland, OH) panels and interpreted according to the Clinical and Laboratory Standards Institute (CLSI) guidelines.

Results

A total of 717 *Candida* bloodstream isolates were identified. The most common species were *Candida albicans (C. albicans)* (242/717; 33.8%), *Nakaseomyces glabratus (N. glabratus) *(230/717; 32.1%), *C. parapsilosis* (122/717; 17.0%), *C. tropicalis* (56/717; 7.8%), *C. dubliniensis* (30/717; 4.2%), *Pichia kudriavzevii (P. kudriavzevii)* (15/717; 2.1%), and *Clavispora lusitaniae (C. lusitaniae)* (13/717; 1.8%). Overall, non-*albicans *species accounted for 475 of 717 cases (66.2%). Antifungal susceptibility remained largely preserved during most of the study period. *C. albicans* showed low resistance to fluconazole (5/242; 2.1%) and voriconazole (1/242; 0.4%), with no echinocandin resistance detected. Among *N. glabratus*, 23 (10%) of 230 isolates were fluconazole-resistant, and echinocandin resistance was uncommon. *C. parapsilosis* and *C. tropicalis* demonstrated moderate fluconazole resistance. Resistance increased in 2025, particularly to fluconazole (13/79; 16.5%) and caspofungin (9/79; 11.4%).

Conclusions

Non-*albicans *species caused most candidemia cases, with *N. glabratus* occurring nearly as often as *C. albicans*. Emerging resistance underscores the importance of species-level identification, susceptibility testing, and ongoing surveillance to guide therapy and antifungal stewardship.

## Introduction

*Candida* species represent a major cause of invasive fungal disease, particularly bloodstream infections, in hospitalized patients worldwide. Candidemia, the most frequent manifestation of invasive candidiasis, is largely healthcare-associated and occurs in both nosocomial and early community-onset settings [1]. These infections are linked to significant morbidity, prolonged hospitalization, increased healthcare expenditures, and notably high crude mortality rates. Common predisposing factors include exposure to broad-spectrum antibiotics, immunosuppressive therapies, renal replacement therapy, and intravascular devices [2].

*Candida albicans (C. albicans)* remains the most common cause of candidemia worldwide; however, a substantial shift toward non‑albicans *Candida* (NAC) species has emerged over recent decades. In the United States, population‑based studies show a decline in *C. albicans* bloodstream infections and an increasing prevalence of *Nakaseomyces glabratus (N. glabratus)*, *C. parapsilosis*, *C. tropicalis*, and Pichia *kudriavzevii (P. kudriavzevii)*. This shift is clinically significant because several NAC species exhibit reduced susceptibility to fluconazole and inherent limitations to echinocandin therapy, particularly for *C. parapsilosis* [3,4].

Antifungal susceptibility testing of *Candida* isolates has become a critical component of epidemiologic surveillance, enabling the detection of resistance patterns and temporal trends through standardized methodologies. Surveillance studies have identified rising resistance to fluconazole among certain NAC species, including *C. parapsilosis*, *Meyerozyma guilliermondii (M. guilliermondii)*, *Clavispora lusitaniae (C. lusitaniae)*, and *N. glabratus*, while susceptibility to echinocandins has generally remained stable, though emerging resistance has been reported [5]. Changes in antifungal use, including widespread prophylaxis and empiric therapy, may have contributed to shifts in species distribution and resistance patterns.

Because species prevalence and susceptibility vary by region and institution, ongoing local surveillance and species-level identification are critical to ensuring effective therapy and supporting antifungal stewardship [6]. The aim of this study was to characterize the epidemiology and antifungal susceptibility patterns of *Candida* species isolated from blood cultures at a tertiary care academic medical center between 2018 and 2025. By evaluating species distribution and resistance profiles to commonly used antifungal agents, this study sought to provide updated local surveillance data to inform empirical treatment decisions and support antifungal stewardship efforts.

This work was previously presented as a meeting abstract at the ASM Microbe 2026 Annual Meeting on June 6, 2026.

## Materials and methods

Study design and setting

All *Candida* isolates recovered from positive blood cultures obtained at Atrium Health Wake Forest Baptist between January 1, 2018, and December 31, 2025, were eligible for inclusion. A candidemia case was defined as the isolation of a *Candida* species from at least one blood culture collected from a patient with clinical findings consistent with bloodstream infection. To avoid overrepresentation of repeated cultures from the same infection event, only the first bloodstream isolate from the first candidemia episode for each patient was included in the analysis. Repeat blood culture isolates yielding the same *Candida* species from the same patient during an ongoing infection episode were considered duplicates and excluded.

Because only the first candidemia episode per patient was analyzed, each patient contributed a single isolate to the study dataset. Additional exclusion criteria included isolates lacking definitive species-level identification, isolates without antifungal susceptibility testing results, and non-bloodstream *Candida* isolates recovered from other specimen sources. The study was approved by the Atrium Health Wake Forest Baptist Institutional Review Board (IRB No. IRB00150042). Patient identifiers were removed prior to analysis to maintain confidentiality.

Isolate collection and identification

A total of 717 non-duplicate *Candida* isolates recovered from bloodstream infections during the study period, along with their corresponding antifungal susceptibility results, were retrospectively analyzed. Blood cultures were collected as part of routine clinical care and incubated using the BD BACTEC™ FX automated blood culture system (Becton Dickinson, Franklin Lakes, NJ) with aerobic, anaerobic, and mycosis blood culture bottles according to the manufacturer's recommendations and routine laboratory protocols.

When a blood culture bottle signaled positive, an aliquot of broth was aseptically removed and subjected to Gram staining. Specimens demonstrating yeast cells on Gram stain were subcultured onto Sabouraud Dextrose Agar (Hardy Diagnostic, Santa Maria, CA) plates and incubated aerobically at 30°C for 24-48 hours. Culture plates were examined daily for growth, and well-isolated colonies were selected for identification.

Species identification was performed using matrix-assisted laser desorption/ionization time-of-flight mass spectrometry (MALDI-TOF MS) with the Bruker MALDI Biotyper system (Bruker Daltonics, Bremen, Germany) according to the manufacturer's instructions. Identification results were accepted when confidence scores met the manufacturer's recommended criteria for species-level identification. When necessary, isolates were retested to confirm identification results [6,7].

Antifungal susceptibility testing

Antifungal susceptibility testing was performed using Sensititre™ YeastOne™ YO9 or Sensititre™ YeastOne™ YO11 colorimetric broth microdilution panels (Thermo Fisher Scientific, Cleveland, OH) according to the manufacturer's instructions. Prior to clinical implementation, the Sensititre™ YeastOne™ system was validated in our laboratory against the Clinical and Laboratory Standards Institute (CLSI) reference broth microdilution method and demonstrated acceptable performance characteristics. Before antifungal susceptibility testing, isolates were subcultured onto Sabouraud Dextrose Agar to ensure purity and viability. Fresh yeast colonies were used to prepare standardized inocula according to the manufacturer's recommendations. The inoculated Sensititre™ YeastOne™ plates were incubated at 35°C and examined after 24 hours of incubation, with additional incubation up to 48 hours when necessary for slow-growing isolates.

Minimum inhibitory concentrations (MICs) were determined by visual assessment of colorimetric changes using a plate-reading mirror. Growth was indicated by a color change from blue to red resulting from metabolic reduction of the indicator dye incorporated into the medium. The MIC endpoint for each antifungal agent was defined as the lowest drug concentration that prevented the color change to red, corresponding to the first well remaining blue [8]. The antifungal agents evaluated included fluconazole, voriconazole, posaconazole, anidulafungin, micafungin, caspofungin, and amphotericin B, depending on panel configuration.

MIC results were interpreted according to CLSI M27M44S standards using species-specific clinical breakpoints when available. Isolates were categorized as susceptible, susceptible-dose dependent/intermediate, or resistant using CLSI clinical breakpoints when available. For organism-antifungal combinations without CLSI clinical breakpoints, interpretation was based on CLSI epidemiologic cutoff values (ECVs) [9]. Quality control was performed with each lot and testing period using the CLSI-recommended reference strains *Candida parapsilosis* ATCC 22019 and *Pichia kudriavzevii* ATCC 6258. All quality control MIC results were within the established CLSI acceptable ranges throughout the study period [9].

Statistical analysis

Descriptive statistics were used to summarize patient demographics, species distribution, and antifungal susceptibility data. Categorical variables were reported as frequencies and percentages, whereas continuous variables were summarized as medians and ranges. MIC distributions were summarized using MIC₅₀ and MIC₉₀ values. All statistical analyses were performed using Microsoft Excel (Microsoft Corp., Redmond, WA).

## Results

The 717 unique patients from whom *Candida* strains were isolated had a median age of 52 years and a mean age of 51.9 years (range: 1-88 years). Males accounted for 415 cases (57.9%). The highest proportion of *Candida* isolates originated from patients aged > 65 years (253/717; 35.3%), followed by individuals aged 45-64 years (239/717; 33.3%) and 20-44 years (164/717; 22.9%). In contrast, markedly lower proportions were observed in younger age groups, including infants < 1 year (38/717; 5.3%) and children and adolescents aged 1-19 years (23/717; 3.2%) (Table 1).

**Table 1:** Demographic characteristics of patients with candidemia (2018-2025)

Characteristics	Values
Patient demographics	(N = 717)
Sex, n (%)	
Male	415 (57.9)
Female	302 (42.1)
Median age, years (range); mean age, years (range)	52 (1-88); 51.9 (1-88)
Age group, years, n (%)	
< 1	38 (5.3)
1-19	23 (3.2)
20-44	164 (22.9)
45-64	239 (33.3)
> 65	253 (35.3)

Overall, *C. albicans* was the most common species (242/717; 33.8%), followed by *N. glabratus* (230/717; 32.1%) and *C. parapsilosis* (122/717; 17.0%). *C. tropicalis* accounted for 56 of 717 isolates (7.8%), while less frequently identified species included *C. dubliniensis* (30/717; 4.2%), *P. kudriavzevii* (15/717; 2.1%), and *C. lusitaniae* (13/717; 1.8%). Other *Candida* species represented 1.3% of all isolates (9/717) and included *Candidozyma auris*, *Candida catenulata*, *M. guilliermondii*, *Candida intermedia*, *Yarrowia lipolytica*, *Nakaseomyces nivariensis*, and *Candida zeylanoides* (Table 2). Candidemia was more frequently caused by NAC species (475/717; 66.2%) than by *C. albicans* (242/717; 33.8%).

**Table 2:** Species distribution of Candida isolates from candidemia cases (2018-2025) ^*^Candidozyma auris (n = 2), *Candida catenulata *(n = 2), *Meyerozyma guilliermondii *(n = 1), *Candida intermedia *(n = 1), *Yarrowia lipolytica *(n = 1), *Nakaseomyces nivariensis *(n = 1), and *Candida zeylanoides* (n = 1)

Organisms	Overall		2018		2019		2020		2021		2022		2023		2024		2025	
	N	%	N	%	N	%	N	%	N	%	N	%	N	%	N	%	N	%
Candida albicans	242	33.8	25	30.2	37	41.2	26	33.8	37	34.6	28	30.3	33	32.1	28	32.5	28	35.4
Nakaseomyces glabratus	230	32.1	30	36.1	20	22.2	28	36.3	32	29.8	30	32.5	27	26.2	32	37.2	31	39.2
Candida parapsilosis	122	17.0	12	14.5	16	17.8	12	15.6	14	13.1	14	15.1	23	22.3	22	25.6	9	11.4
Candida tropicalis	56	7.8	7	8.4	10	11.1	5	6.5	9	8.4	7	7.5	10	9.7	2	2.3	6	7.6
Candida dubliniensis	30	4.2	6	7.2	3	3.3	4	5.2	5	4.7	3	3.3	8	7.8	1	1.2	0	0
Pichia kudriavzevii	15	2.1	1	1.2	2	2.2	1	1.3	5	4.7	2	2.2	2	1.9	1	1.2	1	1.3
Clavispora lusitaniae	13	1.8	1	1.2	1	1.1	1	1.3	2	1.9	6	6.4	0	0	0	0	2	2.6
Other*	9	1.3	1	1.2	1	1.1	0	0	3	2.8	2	2.2	0	0	0	0	2	2.6
Total	717		83		90		77		107		92		103		86		79	

Table 3 provides susceptibility data for the four most common *Candida* species. Antifungal susceptibility testing was also performed for less common species; however, these results are not shown because of the limited number of isolates. Across all study years, *C. albicans* isolates remained uniformly susceptible to amphotericin B and echinocandins, with low and stable MIC₅₀/MIC₉₀ values, while fluconazole and voriconazole susceptibility exceeded 92% overall, despite occasional year‑to‑year variability. *N. glabratus* demonstrated high rates of fluconazole susceptible‑dose-dependent (SDD) categorization across all years, while variable susceptibility to voriconazole and posaconazole was observed over time.

**Table 3:** In vitro susceptibilities of Candida isolates (2018-2025) I = intermediate; S = susceptible; R = resistant; NA = not applicable; SDD = susceptible-dose-dependent (applicable only to fluconazole according to CLSI species-specific breakpoints). For agents without CLSI clinical breakpoints (amphotericin B, posaconazole, and voriconazole against *Nakaseomyces glabratus*), values in the “S” column represent wild-type isolates based on CLSI epidemiologic cutoff values, not clinical susceptibility MIC: minimum inhibitory concentration; CLSI: Clinical and Laboratory Standards Institute

Organisms	Antifungal name	Overall					2018			2019			2020			2021			2022			2023			2024			2025		
		I/SDD	S	R	MIC₅₀, µg/mL	MIC₉₀, µg/mL	I/SDD	S	R	I/SDD	S	R	I/SDD	S	R	I/SDD	S	R	I/SDD	S	R	I/SDD	S	R	I/SDD	S	R	I/SDD	S	R
Candida albicans	Amphotericin B	NA	100	NA	0.5	1	NA	100	NA	NA	100	NA	NA	100	NA	NA	100	NA	NA	100	NA	NA	100	NA	NA	100	NA	NA	100	NA
	Fluconazole	1.2	96.7	2.1	0.5	1	0	96	4	2.7	94.6	2.7	0	96.2	3.8	2.7	97.3	0	0	100	0	0	96.9	3.1	0	100	0	3.6	92.8	3.6
	Voriconazole	2.5	97.1	0.4	0.015	0.03	4	96	0	2.7	94.6	2.7	3.8	96.2	0	2.7	97.3	0	0	100	0	0	96.9	3.1	0	100	0	3.6	96.4	0
	Posaconazole	NA	92.9	NA	0.03	0.06	NA	92	NA	NA	94.6	NA	NA	88.4	NA	NA	89.2	NA	NA	96.4	NA	NA	96.9	NA	NA	96.4	NA	NA	89.3	NA
	Caspofungin	0	100	0	0.06	0.12	0	100	0	0	100	0	0	100	0	0	100	0	0	100	0	0	100	0	0	100	0	0	100	0
	Micafungin	0	100	0	0.015	0.015	0	100	0	0	100	0	0	100	0	0	100	0	0	100	0	0	100	0	0	100	0	0	100	0
	Anidulafungin	0	100	0	0.03	0.12	0	100	0	0	100	0	0	100	0	0	100	0	0	100	0	0	100	0	0	100	0	0	100	0
Nakaseomyces glabratus	Amphotericin B	NA	100	NA	1	1	NA	100	NA	NA	100	NA	NA	100	NA	NA	100	NA	NA	100	NA	NA	100	NA	NA	100	NA	NA	100	NA
	Fluconazole	90	NA	10	8	32	86.7	NA	3.3	80	NA	20	82.1	NA	0	93.8	NA	0	100	NA	0	96.3	NA	3.7	100	NA	0	77.4	NA	22.6
	Voriconazole	NA	53.1	NA	0.25	1	NA	50	NA	NA	45	NA	NA	35.7	NA	NA	59.4	NA	NA	80	NA	NA	59.3	NA	NA	71.9	NA	NA	19.4	NA
	Posaconazole	NA	76.1	NA	1	2	NA	73.3	NA	NA	70	NA	NA	67.9	NA	NA	84.4	NA	NA	93.3	NA	NA	88.9	NA	NA	87.5	NA	NA	41.9	NA
	Caspofungin	6.9	87.4	5.7	0.06	0.25	6.6	90	3.4	10	90	0	7.2	92.8	0	0	100	0	0	100	0	0	88.8	11.2	12.5	84.4	3.1	19.4	54.8	25.8
	Micafungin	0	98.3	1.7	0.015	0.03	0	100	0	0	100	0	0	100	0	0	100	0	0	100	0	0	92.6	7.4	0	100	0	0	96.7	3.3
	Anidulafungin	0.4	98.3	1.3	0.03	0.12	0	100	0	0	100	0	0	100	0	0	100	0	0	100	0	0	92.3	7.7	0	100	0	3.2	90.3	6.5
Candida parapsilosis	Amphotericin B	NA	96.6	NA	0.5	1	NA	100	NA	NA	100	NA	NA	96.2	NA	NA	100	NA	NA	100	NA	NA	100	NA	NA	95.5	NA	NA	87.5	NA
	Fluconazole	3.3	90.1	6.6	1	4	0	90	10	0	100	0	0	100	0	0	93.3	6.7	0	92.9	7.1	0	81.8	18.2	9.1	86.4	4.5	22.2	77.8	0
	Voriconazole	6.8	91.5	1.7	0.015	0.12	10	90	0	0	100	0	0	100	0	13.3	86.7	0	14.3	85.7	0	4.6	86.4	9	4.5	94.5	1	11.1	88.9	0
	Posaconazole	NA	96.6	NA	0.06	0.12	NA	100	NA	NA	100	NA	NA	100	NA	NA	100	NA	NA	100	NA	NA	86.4	NA	NA	95.5	NA	NA	100	NA
	Caspofungin	0	100	0	0.5	1	0	100	0	0	100	0	0	100	0	0	100	0	0	100	0	0	100	0	0	100	0	0	100	0
	Micafungin	2.5	97.5	0	1	2	0	100	0	0	100	0	9.1	90.9	0	6.7	93.3	0	0	100	0	0	100	0	0	100	0	12.5	87.5	0
	Anidulafungin	0.8	99.2	0	1	2	0	100	0	0	100	0	0	100	0	0	100	0	0	100	0	0	100	0	0	100	0	12.5	87.5	0
Candida tropicalis	Amphotericin B	NA	98.2	NA	1	2	NA	100	NA	NA	100	NA	NA	100	NA	NA	100	NA	NA	100	NA	NA	100	0	NA	100	NA	NA	83.3	NA
	Fluconazole	14.3	76.8	8.9	2	4	16.7	83.3	0	10	80	0	16.7	83.3	0	22.2	77.8	0	0	100	0	20	70	10	0	100	0	16.7	50	33.3
	Voriconazole	19.6	73.2	7.2	0.12	0.5	16.7	83.3	0	40	60	0	50	50	0	22.2	77.8	0	0	100	0	10	80	10	0	100	0	0	50	50
	Posaconazole	NA	50	NA	0.25	0.5	NA	50	NA	NA	40	NA	NA	50	NA	NA	66.7	NA	NA	50	NA	NA	40	NA	NA	100	NA	NA	0	NA
	Caspofungin	0	98.2	1.8	0.06	0.25	0	100	0	0	100	0	0	100	0	0	100	0	0	100	0	0	100	0	0	100	0	0	83.3	16.7
	Micafungin	0	98.2	1.8	0.03	0.06	0	100	0	0	100	0	0	100	0	0	100	0	0	100	0	0	100	0	0	50	50	0	100	0
	Anidulafungin	0	98.2	1.8	0.06	0.25	0	100	0	0	100	0	0	100	0	0	100	0	0	100	0	0	100	0	0	100	0	0	83.3	16.7

Echinocandin activity against *N. glabratus* remained high overall; however, a decline in caspofungin susceptibility was observed in 2025. Given the relatively small number of isolates tested that year, this finding should be interpreted with caution. For *Candida parapsilosis*, amphotericin B and azole susceptibility remained high overall; however, consistently higher echinocandin MICs were observed compared with other species, particularly for caspofungin and micafungin, with modest year‑to‑year variation. *Candida tropicalis* exhibited generally high susceptibility to echinocandins and amphotericin B, while azole susceptibility showed greater interannual variability (Table 3).

## Discussion

Candidemia causes approximately 22,000 infections annually in the United States and remains one of the most common healthcare‑associated bloodstream infections. Although incidence declined after the early 2000s, more recent surveillance demonstrates a plateau and stabilization between 2009 and 2017 across US sites [10]. While more than 100 *Candida* species have been described, only a limited number are clinically relevant. *C. albicans* remains the most frequently isolated species globally, accounting for approximately 40-70% of cases, although species distribution varies by institution and region [11,12]. In this retrospective study spanning the period from 2018 to 2025, *C. albicans* (33.8%) was the most common species, closely followed by *N. glabratus* (32.1%).

This near‑equal distribution is consistent with recent Centers for Disease Control and Prevention (CDC) Emerging Infections Program data, which report *C. albicans* accounting for 37-39% of candidemia cases and *N. glabratus* for 28-30% [3,13,14]. Similar findings from Atlanta and Baltimore further demonstrate that NAC species collectively cause the majority of candidemia cases [5,15]. These results align with US reports documenting the increasing prominence of *N. glabratus*, including population‑based surveillance from Alameda County, California, where *N. glabratus* equaled or exceeded *C. albicans* between 2017 and 2020 [1]. Historically, *C. albicans* accounted for over 40-50% of cases in earlier US studies [2,16]. Geographic variation persists, with *C. albicans* remaining more common in Northern and Central Europe but less dominant in Southern Europe, Latin America, Australia, and the US [17,18]. The narrowing difference observed in our study, along with year‑to‑year variability, underscores the increasing clinical importance of *N. glabratus* and the need for continued local surveillance.

In our study, candidemia rates were higher among males (57.9%) than females, consistent with multiple US studies reporting higher rates in men across different surveillance periods and healthcare settings [2,3,13,14]. Rates were highest among patients aged > 65 years, followed by those aged 45-64 years, mirroring national observations that candidemia disproportionately affects older adults [1,13,14]. In contrast, pediatric cases were uncommon, reflecting recent US trends outside the neonatal population [3,19].

Overall antifungal activity remained largely preserved throughout the study period. *C. albicans* demonstrated uniform susceptibility to amphotericin B and echinocandins, with fluconazole and voriconazole susceptibility exceeding 92%, consistent with prior US surveillance data reporting fluconazole resistance rates of ≤ 2% [5,16,19]. Despite modest changes in fluconazole and voriconazole susceptibility over time, categorical resistance remained rare, supporting the continued effectiveness of azoles and echinocandins for *C. albicans* candidemia in the *United States* setting.

As expected, *N. glabratus* exhibited reduced susceptibility to triazoles, with a high proportion of isolates categorized as fluconazole SDD, paralleling long‑standing national trends reporting resistance or SDD rates of approximately 8-20% [3,5,13,19]. Echinocandin activity remained high overall; however, increasing intermediate rates for caspofungin were observed in later years, consistent with reports of echinocandin resistance in US surveillance studies [13,15].

For *C. parapsilosis*, amphotericin B and azole susceptibility remained high, while echinocandin MICs were consistently higher than those observed for other species, reflecting intrinsic reduced susceptibility, with categorical resistance remaining uncommon [3,5,13]. *C. tropicalis* demonstrated preserved susceptibility to echinocandins and amphotericin B, with more variable azole susceptibility over time, a pattern consistent with national data reporting low but fluctuating azole resistance rates [3,5,13,19]. Overall, susceptibility rates remained high for most antifungal agents across the major *Candida* species, supporting their continued clinical utility while highlighting the importance of ongoing local surveillance to monitor resistance trends.

Our eight‑year institutional experience demonstrates antifungal susceptibility patterns that are broadly concordant with US population‑level data, while providing contemporary, granular insights into MIC trends, particularly for *N. glabratus* and echinocandin activity. The observed stability of susceptibility among *C. albicans* and the differences in MIC distributions among NAC species highlight the importance of routine susceptibility testing to guide therapy, support antifungal stewardship, and monitor local susceptibility patterns.

This study has several limitations. Firstly, the retrospective, single-center design may limit the generalizability of the findings. Second, the analysis was descriptive and did not include inferential statistical testing to assess the significance of temporal changes in antifungal susceptibility. Third, annual isolate numbers were small for some species, particularly in later years, which may limit the reliability of year-to-year comparisons and resistance trend analyses. In addition, detailed clinical information, antifungal treatment data, and patient outcomes were not available for analysis. Finally, several less common *Candida* species, including *C. auris*, were represented by only a small number of isolates, which limited meaningful analysis of species-specific antifungal susceptibility patterns and resistance trends.

Despite these limitations, the large number of bloodstream isolates analyzed over eight years provides valuable contemporary data on local candidemia epidemiology and antifungal susceptibility patterns. This study demonstrates that NAC species predominate in contemporary candidemia at our institution, with species distribution and antifungal susceptibility patterns closely paralleling recent US surveillance data. Although overall antifungal activity remains largely preserved, species-specific differences in MIC distributions, particularly among *N. glabratus*, highlight the ongoing need for species-level identification and routine antifungal susceptibility testing to guide empiric therapy and support antifungal stewardship efforts.

## Conclusions

This eight‑year retrospective analysis provides updated institutional data on the epidemiology and antifungal susceptibility patterns of *Candida* species causing bloodstream infections at a large tertiary care academic medical center. NAC species accounted for most candidemia cases, with *N. glabratus* nearly equaling *C. albicans* as the most frequently isolated species. Overall antifungal susceptibility remained favorable, particularly for echinocandins and amphotericin B. However, clinically relevant species-specific differences were observed, including reduced azole susceptibility among *N. glabratus* and higher echinocandin MICs for *C. parapsilosis*. These findings highlight the importance of species-level identification and routine antifungal susceptibility testing to guide appropriate therapy. Continued local surveillance is needed to monitor species distribution and susceptibility profiles over time, support antifungal stewardship efforts, and optimize the management of candidemia in healthcare settings.
